# Tommy’s Clinical Decision Support Tool: an intervention development and feasibility study to inform a future randomised controlled trial

**DOI:** 10.1186/s40814-026-01788-9

**Published:** 2026-02-25

**Authors:** Jenny Carter, Dilly Anumba, Christy Burden, Siobhán Gillespie, Amy Howell, Andrew Judge, Erik Lenguerrand, Samantha Pérez Amack, Nicola Savory, Elaine Sheehan, Basky Thilaganathan, Maria Viner, Hannah Wilson, Cathy Winter, Jane Sandall, Tommy’s National Centre for Maternity Improvement, Lia Brigante, Carly Edwards, Birte Harlev-Lam, Dorian Martinez, Fran McNeil, Jo Tanner, Gemma Thurston, Dan Wolstenholme

**Affiliations:** 1 Department of Women and Children’s Health, School of Life Course and Population Sciences, King’s College London, 10th Floor, North Wing, St Thomas’ Hospital, Westminster Bridge Road, London, SE1 7EH UK; 2 Tommy’s National Centre for Maternity Improvement, Royal College of Obstetricians and Gynaecologists/Royal College of Midwives, 10‑18 Union Street, London, SE1 1SZ UK; 3 School of Medicine and Population Health, University of Sheffield, The Jessop Wing, Tree Root Walk, Sheffield, S10 2SF UK; 4 Academic Women’s Health Unit, Bristol Medical School, University of Bristol, Southmead Hospital, Bristol, BS10 5NB UK; 5 Bristol Medical School, Translational Health Sciences, University of Bristol, Southmead Hospital, Bristol, BS10 5NB UK; 6 NIHR Bristol Biomedical Research Centre, University Hospitals Bristol and Weston NHS Foundation Trust and University of Bristol, Bristol, UK; 7 Maternal Medicine Department, St George’s University Hospitals NHS Foundation Trust, Blackshaw Road, London, SW17 0QT UK; 8 Cardiovascular and Genomics Research Institute, City St George’s, University of London, Cranmer Terrace, London, SW17 0QT UK; 9 Fetal Medicine Unit, St George’s University Hospitals NHS Foundation Trust, Blackshaw Road, London, SW17 0QT UK; 10 Mothers for Mothers, The Gatehouse Centre, Hareclive Rd, Bristol, BS13 9JN UK; 11 Lewisham and Greenwich NHS Trust, University Hospital Lewisham, Lewisham High Street, London, SE13 6LHUK UK; 12 PROMPT Maternity Foundation, Department of Women’s Health, The Chilterns, Southmead Hospital, Bristol, BS10 5NB UK

**Keywords:** Pregnancy, Risk assessment, Decision support, Clinical Decision Support Systems (CDSS), Medical device, Maternity care

## Abstract

**Background:**

The Tommy’s Clinical Decision Support Tool is a web-based application that is used to assess risk of preterm birth and placental dysfunction. Utilising validated algorithms and rule engines, which are more accurate than current checklist methods, the Tool instantly recommends best evidenced-based care pathways. This personalisation of assessment and decision support could reduce preterm birth and stillbirth, whilst also addressing variation in care. This study aimed to develop the intervention and assess feasibility of implementation in four NHS maternity services to inform a planned cluster randomised controlled trial. We aimed to investigate barriers and facilitators to implementation; reach (whether particular groups are excluded and why), fidelity (degree to which the intervention is delivered as intended), and unintended consequences.

**Methods:**

The NASSS framework (Non-adoption or Abandonment of technology by individuals and difficulties achieving Scale-up, Spread and Sustainability) informed analysis. We used online surveys, semi-structured interviews and focus groups to investigate maternity service user and healthcare professional (HCP) experience.

**Results:**

One thousand one hundred eighty-one maternity service users and 112 HCPs participated, completing 1260 online surveys, 8 focus groups and 29 semi-structured interviews (women: n = 24; HCPs: n = 23). Overall, the Tool appears acceptable and easy-to-use for both pregnant and HCP users, although the burden of introducing a novel intervention within an already overstretched service was identified as a potential barrier to successful implementation. Findings influenced developments of the device and implementation strategy ahead of the trial. Lessons learned highlighted the importance of: availability of the Tool to guide care for all, including pregnant users unable, or choosing not to engage with it; top-level and multidisciplinary buy-in; dedicated resources; preparation for transitional period; local champions across professions and settings; clarity in purpose, scope, potential benefits and evidence-base; mitigation of double data entry; IT infrastructure optimisation; flexibility in training and accessibility of implementation resources. Further refinements will include non-English translation of the pregnant user interface.

**Conclusions:**

Tommy’s Tool has the potential to make providing optimal maternity care easier for health professionals, which could reduce variation in care and ultimately improve outcomes. This study gave us the opportunity to evaluate implementation processes and identify potential barriers to successful implementation. By addressing these barriers, ahead of the trial, we have maximised the chance of the trial results being conclusive.

**Trial registration:**

this study was prospectively registered on ISRCTN:ID13498237, on 31/01/2022.

**Supplementary Information:**

The online version contains supplementary material available at 10.1186/s40814-026-01788-9.

## Key messages regarding feasibility

- Ahead of a cluster randomised controlled trial, it was important to identify and manage potential barriers to successful implementation of this novel complex intervention.
- Our findings highlighted: a need for clarity about purpose, scope and potential benefits; multidisciplinary buy in from the start will aid smooth implementation; that novel interventions that are system-wide require preparation for a transitional period; dedicated resources should include a paid project lead, preferably a midwife, with admin support; local champions across settings, not just professions; digital interventions require optimisation of the hospital’s IT infrastructure; flexibility in training and accessibility of resources are required.
- This study gave us the opportunity to optimise the intervention and the implementation strategy, as well as refining the RCT protocol, which will increase the chances of the trial findings being meaningful and conclusive.

## Background

The Tommy’s National Centre for Maternity Improvement (TNCfMI) developed the Tommy’s Pathway: a Clinical Decision Support Tool, to reduce rates of preterm birth and stillbirth, and variation in care. This web-based clinical decision support tool aims to provide assessments of risk for preterm birth and placental dysfunction more accurately than current methods and recommends evidenced-based care pathways in a format accessible to both women and healthcare professionals (HCPs).

Successful implementation of a healthcare intervention in one context is not necessarily replicated in others [1]. Therefore, we adopted a scientific evidence-based approach to optimising the conditions for successful implementation of a complex intervention at scale. This paper reports findings from an implementation evaluation that was carried out in four NHS organisations where the intervention was to be implemented as a service development project. The aim of this study was to inform a forthcoming cluster randomised controlled trial, PARTNER: A Clinical Decision Support Tool To Reduce Placental Disorders and Preterm birth in Pregnancy Trial. This trial is needed because although the algorithms and rule engines have been evaluated in research settings, they have not been rigorously tested in the “real world”, or all together as part of this complex intervention. Study objectives were to evaluate: acceptability and usability of the Tool to women and HCPs; barriers to and facilitators of implementation; reach (i.e. the extent to which the Tool was used within the intended population); fidelity of the Tool and implementation strategy, and unintended consequences.

The estimated sample size for evaluating overall fidelity—defined as the proportion of service users registered on the Tool—was based on 16,653 births, reflecting the average number recorded in NHS Digital’s National Maternity Dataset between December 2020 and November 2021. Based on birth numbers across participating Trusts, and assuming a 20% response rate, we estimated that approximately 3,330 women would complete the online survey over a nine-month period, starting three months after the Tool was launched at each site. From our survey sample, we aimed to recruit a purposive sample of 50 maternity service users who would be invited to take part in interviews and focus groups. This sample size was considered achievable within the project timeframe and sufficient to generate meaningful insights and achieve data saturation. For this feasibility study, we sought to capture feedback from as diverse a sample of maternity service users as possible in terms of demographics and care pathways.

Acceptability and usability are clearly important aspects of successful implementation and will be reported elsewhere. Reach, and the potential of the Tool to address disparities in care provision, will be the focus of another paper. In this paper, we report the specific implementation outcomes: barriers and facilitators, fidelity, unintended consequences, and the practical adaptations put in place to improve the Tool and implementation strategy ahead of the RCT.

HRA REC approval was granted by the London – Bromley Research Ethics Committee (REC) (IRAS 294819; REC Ref 21/PR/1029) and the study was prospectively registered on ISRCTN, ID 13498237. A REC substantial amendment was later approved which included: extending the period for recruitment, the addition of antenatal and HCP online questionnaires and the use of posters and text messaging to increase and widen participation. A detailed description of the Tool and its development, guided by the TIDieR checklist [2] has been reported previously, along with the protocol for this study [3], but a summary is reported below.

### Development of the Tool

**Fig. 1:**
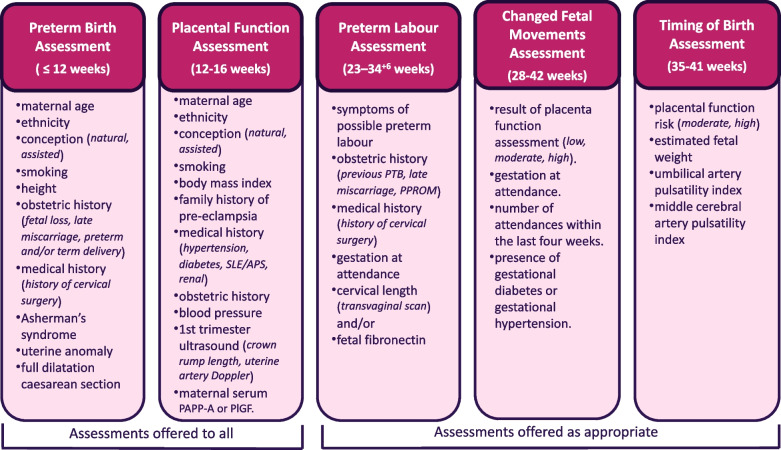
Tommy's Tool Assessments and factors included in algorithm or rule engine

The TNCfMI team worked with the Belgium based software developer, Imec, to create the Tool. This involved developing and refining a software plan in collaboration with a multidisciplinary “Community of Practice”, comprising professionals and maternity service users with lived experience, through a series of workshops between November 2019 and September 2020. This covered Tool features, parameters and criteria on which the selection of assessment algorithms was based. Further development and refinement workshops followed release of the “minimal viable product” (MVP), after which the final application (v1.0) was available for release in August 2021. The Tool offers five risk assessments with associated recommended care pathways aligned to national clinical guidance. The first two, assessing risk of preterm birth and placental dysfunction, are offered to all maternity service users. Other assessments are offered to women experiencing symptoms of possible preterm labour or changes in fetal movements, and a timing of birth assessment is offered to those at moderate or high risk of placental dysfunction. The factors included in assessment algorithms or rule engines are shown in Fig. 1.

### Implementation of the Tool

**Fig. 2:**
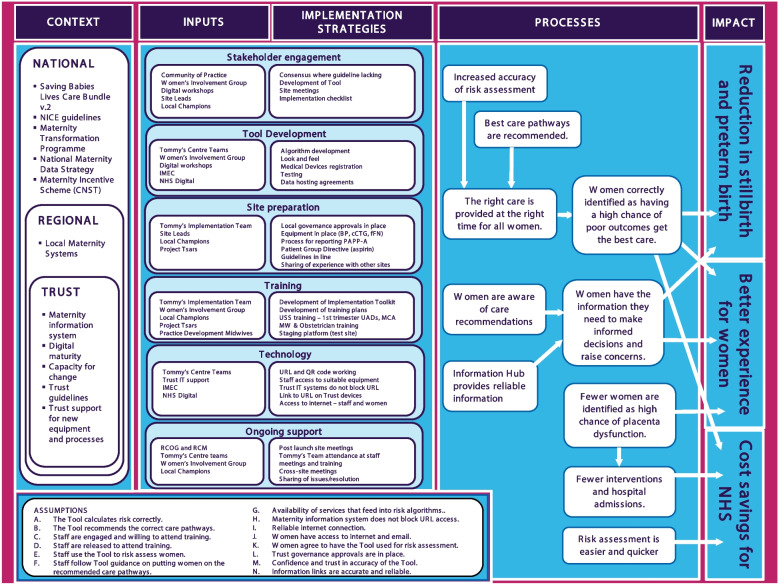
Tommy's Clinical Decision Support Tool Implementation Evaluation Theory of Change Map

The initial implementation strategy was based on a theory of change analysis (Fig. 2) and consideration of important factors to maximise the chances of successful implementation, which are: a strong evidence base; professional consensus; service user and provider involvement; adequate training for clinicians; decision support results available to healthcare service users as well as providers; automatic provision of decision support and provision of decision support where and when decisions are being made [4].

Regular pre-launch site meetings were held with local teams and the TNCfMI. A comprehensive checklist was used to support progress towards implementation readiness (Additional File 1). Local champions were “appointed” from each professional group: doctors, midwives and sonographers, who provided onsite support to HCP users.

Staff training was carried out at the discretion of local sites using resources provided by the TNCfMI. These resources were provided as a comprehensive “Implementation Toolkit” which included: background information about the Tool, its algorithms, rule engines and care pathways, key opinion leader videos, patient information sheets, posters, recommended training timeline, standard operating procedures, slides and videos.

Originally, launch of the Tool in all sites was to be in the spring or summer of 2020, but the COVID pandemic caused considerable delay; competing priorities and staff shortages led to a reluctance to burden already over-stretched staff with the challenge of implementing a system-wide service change. Eventually, however, the first two sites (Maternity Services A and B) were launched in September and October 2021, and the third (Maternity Service C) in July 2022. These were based in North England, South East England and London). Regular individual site meetings continued after “go-live” through the early implementation phase (months 1–6) followed by cross-site meetings that enabled shared learning between local site teams as well as ongoing TNCfMI support.

## Methods

Methods for this study have been previously described in detail [3], however, in summary:

### Study Design

This project was an exploratory mixed-methods implementation evaluation study, based on current guidance [5–7], which aimed to provide evidence to inform adaptations to intervention components and implementation strategies in a forthcoming cluster RCT. The study was carried out in four NHS maternity services (five hospital sites). Interviews and focus groups took place between 4th February 2022 and 3rd April 2023. Data collection through the online surveys started on 22nd February 2022 and was completed on 31st March 2023.

### Data collection methods

Participants were eligible if they were receiving or providing maternity care at participating sites. This included those under the age of 16 (whose parent or guardian would be asked to sign consent in addition to the child signing an assent form). Posters and emails were used to invite potential participants to complete an online survey. Posters aimed at maternity services users were placed in patient waiting areas, while posters promoting the study to HCPs were placed in staff areas. Emails were sent to maternity service users who had agreed to contact from researchers when they registered on the Tool. Site Research Midwives forwarded invitation emails to maternity service worker email distribution lists. Quick Response (QR) codes on the posters, and links within the emails, took potential participants to the surveys which were managed through the Qualtrics survey management platform.

The surveys collected information on the women’s and HCP’s views and experiences of using the Tool. Survey respondents were asked to consider further participation in the study. Those willing, and others directly referred to researchers by the site Research Midwives, were selected for interviews or focus groups through a process of purposive sampling. An interview schedule and topic guide were used to ensure relevant in-depth data on views and experiences were collected from a diverse range of participants. Focus group sizes ranged from two to seven participants. In each hospital, one to two focus groups comprised a diverse mixture of maternity services users, and one to two focus groups comprised a variety of HCPs from different professional groups. Consent was implied on completion of online surveys, and written informed consent was obtained prior to interviews and focus groups.

Aggregate reports of demographic characteristics of women registering on the Tool were compared with maternity bookings as reported by NHS England’s publicly available Maternity Services Data Set (MSDS) [8]. Fidelity was evaluated using study data as well as data derived from TNCfMI’s Quality Management System (QMS) reports, which are a requirement of the UK’s Medicines and Healthcare Products Regulatory Agency (MHRA) medical devices regulations.

### Data analysis

Qualitative data were managed through NVivo qualitative data software and analysed using a thematic framework approach. A second researcher analysed 10% of transcripts to ensure validity of the findings. Data analysis was informed by the NASSS (Non-adoption or Abandonment of technology by individuals and difficulties achieving Scale-up, Spread and Sustainability) framework [9]. Statistical software (IBM, SPSS 28.1.1) was used to analyse quantitative questionnaire data. Participant demographic characteristics and risk profiles were explored using descriptive statistics.

## Findings

### Participants

**Fig. 3:**
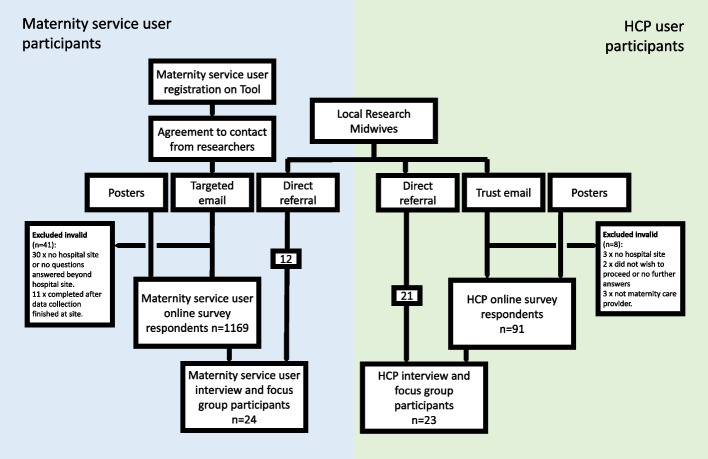
Tommy's Tool Early Adopter Implementation Evaluation participant flow chart

In addition to invitations by posters, which contained QR code links to the online questionnaires, email invitations (and up to 2 reminders) were sent to 1109 potential antenatal participants and 2419 postnatal potential participants. This resulted in response rates of 33.3% (368/1109) and 34.8% (842/2419) respectively. Of the original 368 antenatal online survey respondents, 15 were excluded from analysis: six because no hospital site was provided and 9 because the survey was completed after the site had been closed to data collection. Of the original 842 postnatal survey respondents, 26 were excluded from analysis: 24 because either no hospital had been provided or no questions were answered beyond hospital name, and two because the survey was completed after the site had closed to data collection. This resulted in a total of 1169 maternity service user online survey participants. Participants are summarised in Fig. 3.

Maternity service user participant characteristics are shown in Table 1. A reasonably broad spectrum of participants completed the online survey, with representation across age groups (apart from under 16-year-olds), ethnic groups and areas of social deprivation.

**Table 1:** Demographic characteristics of maternity service user online survey participants

	**(n)**	**(%)**
**NHS TRUST**		
Maternity Service A	586	50.1
Maternity Service B	387	33.1
Maternity Service C	196	16.8
**Total**	**1169**	**100.0**
* Missing*	*0*	
**AGE GROUP**		
< 16	0	
16–19	8	0.9
20–24	61	6.5
25–29	191	20.3
30–34	391	41.6
35–39	241	25.6
40–44	44	4.7
45 +	3	0.3
Would rather not say	1	0.1
**Total**	**940**	**100**
* Missing*	229	
**Ethnicity**		
Asian or Asian British	103	11.0
Black or Black British	47	5.0
Mixed*	30	3.2
Other Ethnic Groups	31	3.3
White	714	76.0
Would rather not say	15	1.6
**Total**	**940**	**100**
* Missing*	229	
Index of Multiple Deprivation (IMD) decile**		
1 (most deprived)	74	13.6
2	40	7.4
3	44	8.1
4	41	7.6
5	55	10.1
6	54	9.9
7	63	11.6
8	54	9.9
9	60	11.0
10 (least deprived)	58	10.7
**Total**	**543**	**100**
* Unknown or Missing*	*626*	

^*^Mixed = a category for individuals who identify with two or more different ethnic backgrounds, as defined by the Office for National Statistics (ONS)

^**^IMD_1 = IMD score falls within 1–10% in UK population, most deprived; IMD_2 = IMD score within 11–20%; IMD_3 = IMD score within 21–30%; IMD_4 = IMD score within 31–40%; IMD_5 = IMD score within 41–50%, IMD_6 = IMD score within 51–60%, IMD_7 = IMD score within 61–60%, IMD_8 = IMD score within 71–80%, IMD_9 = IMD score within 81–90%, IMD_10 = IMD score within 91–100%, least deprived

HCP participant characteristics are shown in Table 2. Most HCP respondents were based in Maternity Service A and C, with only 9 (9.9%) from Maternity Service B. This imbalance was identified early on, and despite efforts to address it, numbers remained low.

**Table 2:** HCP online survey participant characteristics

	**(n)**	**(%)**
**Early Adopter Trust**		
Maternity Service A	45	49.5
Maternity Service B	9	9.9
Maternity Service C	37	40.7
**Total**	**91**	**100.00**
*Missing*	*0*	
**Respondent Profession**		
Midwife	70	76.9
Doctor	18	19.8
Sonographer	3	3.3
**Total**	**91**	**100.0**
* Missing*	*0*	
Work setting*		
Community	36	39.6
Antenatal Clinic	33	36.3
Ultrasound Department	6	6.6
Maternity Assessment or Triage	24	26.4
Early Pregnancy Unit	8	8.8
Labour ward or birth centre	28	30.8
Antenatal Ward	19	20.9
Postnatal Ward	16	17.6
Caseload Team	4	4.4
Other	6	6.6
**Total number of respondents**	**91**	
*HCP could select more than one setting		

Maternity user and HCP interview and focus group participant characteristics are shown in Tables 3 and 4. Three women withdrew after signing consent.

**Table 3:** Maternity service user interview and focus groups participant characteristics

Characteristic		
Maternity Service	n	%
A	12	50.0
B	11	45.8
C	1	4.2
Total	24	
Age group	n	%
16–19	1	4.2
20–24	2	8.3
25–29	4	16.7
30–34	5	20.8
35–39	8	33.3
40–44	4	16.7
Total	24	
Ethnic Group		
Asian Indian	1	4.2
Asian or Asian British	1	4.2
Asian Pakistani	1	4.2
Black African	1	4.2
Mixed	1	4.2
White	3	12.5
White British	10	41.7
White Irish	1	4.2
White Other	5	20.8
Total	24	
IMD decile		
1 (most deprived)	3	18.8
2	1	6.3
3	1	6.3
4	1	6.3
5	0	0.0
6	4	25.0
7	1	6.3
8	2	12.5
9	1	6.3
10 (least deprived)	2	12.5
Total known	16	
Unknown	8

**Table 4:** HCP user interview and focus groups participant characteristics

Characteristic	n	%
NHS Maternity Service		
A	13	56.5
B	4	17.4
C	4	17.4
D	2	8.7
Total	23	
Profession		
Midwife	13	56.5
Doctor (Specialist trainee)	3	13.0
Doctor (Consultant)	5	21.7
Sonographer	1	4.3
Student Midwife	1	4.3
Total	23	
Setting/context		
Clinical Director	1	4.3
Clinical Research Fellow	1	4.3
Community	8	34.8
Co-Project Lead	1	4.3
Co-Transformation Lead	1	4.3
Department Lead	1	4.3
Fetal Medicine Specialist	1	4.3
Hospital Antenatal Clinic	1	4.3
Preterm birth Specialist	1	4.3
Project Lead (current)	1	4.3
Project Lead (former)	1	4.3
Team Leader	1	4.3
Transformation Lead	1	4.3
Work in all areas	3	13.0
Total	23	

### Barriers

#### An additional burden on already overstretched staff

The first two maternity services that launched before June 2022 initially had significant problems with women arriving for appointments without having already registered on Tommy’s Tool. This led to extended visit times and already very busy clinics over-running. The problem appeared to be, largely, a simple lack of awareness:

*“I don't remember anything about this app. even if there was a leaflet…there was so much that you received. I didn't even notice.” [ID-WM003]*

But there was also confusion about how to access the Tool; it was frequently reported that women were looking for “Tommy’s App” on mobile app stores:

*“…quite a lot of women have said they know it's called the Tommy's App, and they've gone on the app store and try to find the app, and it's not an app.” [ID-HCP002]*

*“It's a website with a login, not an app!” [postnatal online survey respondent]*

Better identification of women at risk of preterm birth had also led to an increased demand on hospital preterm specialist services. Some HCPs were also concerned about the additional burden, and potential errors, that could occur during the transitional period. This was because staff had to provide different care for women booked before the launch who were still moving through the system.

#### Change in the aftermath of a global pandemic

The COVID pandemic led to unprecedented high levels of stress on the NHS and its workers. Many service changes had recently been made in response, and the implementation of Tommy’s Tool required another system wide change:

*“I think the biggest issue was with regard to the community midwives...we were already in quite a difficult position with the community midwives basically saying: “Stop, we're overwhelmed. We don't have enough time.” [ID-HCP023]*

Even without the challenge of a global pandemic, any change, not least the implementation of a novel, complex intervention, is likely to create challenges:

*“it's something new, it's uncomfortable, it's another log in, another password.” [ID-HCP026]*

*“you know… five minutes is too much to add on to something that's already really, really busy.” [ID-HCP001].*

Resistance to change appeared to be more pronounced with more experienced/older midwives and senior doctors. Turnover of staff at all levels, including those in managerial or leadership positions, caused delays as new staff needed time to settle in and become engaged with the project.

#### Not all key stakeholders were fully engaged at the start.

Implementing novel interventions requires engagement of all key stakeholders. These stakeholders need to include not just those who are going to authorise and lead the change, but those whose daily working lives will be affected. It became evident that this engagement was not optimal amongst at least one early adopter site:

*“…we could have been better organised at the start... we should have had the head of midwifery, the senior midwifery matrons, the ultrasound departments, maybe even the neonatologists, at the meeting so that it would get that wide departmental buy in, because from the start, it was something that the obstetricians wanted to do, not something that the department wanted to do.” [ID-HCP022]*

#### Expectation that women will register themselves

When the Tool was being developed, it was agreed that women should be encouraged to “take ownership” of it; to register and complete details about themselves and their medical history ahead of their first booking appointment. This approach was strongly influenced by the active involvement of a highly engaged Women’s Advisory Group. Maternity service users were informed about the Tool by email and letters confirming their first appointments, and by posters displayed around the waiting areas. It became apparent, however, that despite efforts to encourage women to engage, there would always be some who were unwilling, or unable to do so. This may have been because they preferred only minimal engagement with health services, or because they did not have access to a smart phone, computer or internet data. HCPs quickly raised concerns that women with limited access to mobile devices and/or data might be excluded from accessing the Tool, and that these women were more likely to be from groups most likely to experience poor pregnancy outcomes. These concerns were addressed with the introduction of the facility allowing HCPs to register women on their behalf.

#### Sub-optimal Information Technology (IT) Infrastructure

There were no major issues with the Tommy’s Tool itself, for example there were zero instances of the website being unavailable or “crashing”. However, there were issues with local hospital IT infrastructure, due specifically to problems with internet access and inadequate devices meaning that hospital records were at times inaccessible to staff working in the community. As one participant reported:

*“The GP said, ‘I can't see your hospital notes’ and you think, well why not?” [ID-WM016]*

Several HCPs reported inadequate, outdated and slow computer equipment, with little hope that the IT infrastructure situation would improve in the foreseeable future:

“… And then the computers are cr*p anyway, and so they take 10 minutes to turn on and all the rest… the IT has always been terrible... I'm not sure it's going to get better.” [ID-HCP001]

#### Training issues

Initially, some midwives felt that the level of training they received was insufficient, and a proportion of HCP survey respondents (13/91, 14.3%) reported having had no training at all:

*“Most of the staff that I speak to feel that there wasn't enough training… they do know how to use it, and they do know what it's for…. But they felt in the beginning that they didn't get enough training.” [ID-HP010].*

Many HCPs, particularly community midwives, didn’t want, or have time for, in-depth training. They just wanted someone to show them how to use it:

*“You don't want to look at slides of it, you just want to have a play with it... I think having training where everybody has to sit in a room, it's ridiculous. Like it's the 21 st century … it should be a more digital solution, it's a digital intervention.” [ID-HCP024]*

These concerns were addressed with the introduction of the Training Platform, a replica website pre-loaded with dummy data ‘blueprints’ to allow HCPs to self-learn using a standalone platform where they can manipulate pre-loaded data to simulate use of the live platform.

### Facilitators

#### Local site Leads and named champions

The commitment and enthusiasm of the local Leads and champions was one of the most important factors in implementation. The onsite support these individuals gave staff was vital to successful implementation. This included raising awareness ahead of launch at staff meetings, running training sessions and providing onsite, one-to-one support in the early weeks after launch. Collaboration with the TNCfMI team ensured that issues arising were addressed quickly, with adaptations made as detailed in Additional files 2, 3 and 4.

The initial plan was to appoint local champions from each professional group, i.e. doctors, midwives and sonographers. This plan was adapted later to expand the cover to include all settings, i.e. antenatal clinic, labour ward and community, as well as professional groups. The process of recruiting and replacing champions was made smoother by the introduction of a document detailing the role of the local champion, which was provided to interested individuals.

#### Tommy’s Centre support

The TNCfMI team provided ongoing support to local early adopter sites during set up and beyond, hosting regular pre and post launch virtual site meetings. The meetings were attended by site leads and champions, members of the TNCfMI team which included ultrasound (senior sonographer) and preterm birth (specialist obstetrician) experts. These experts had been appointed to support early adopter site staff with specific challenges, helping them to identify local resolutions, e.g. a poster outlining different cervical procedures and whether they required referral to specialist clinics, and development of a uterine artery Doppler training programme for new and locum sonographers. The team also supported staff by liaising with departments and individuals e.g. governance, IT.

Informal feedback and processes were put in place to encourage and respond to feedback, in addition to the formal implementation evaluation study. Tommy's Centre Leads, who are respected and well-known leaders in their field, attended meetings and corresponded directly with local clinical leads and influencers.

A full time TNCfMI “practical implementation” midwife was engaged to support sites with training, as well as dealing with issues arising. This role was established to provide additional support to the existing “project midwives for digital development and implementation”, who had pioneered the ‘design’ of the training and implementation plan and provided vital perspective from those with first-hand knowledge and experience of the needs from the predominant users, midwives. The “practical implementation” midwife role brought the practical experience and the additional team resource required to mobilise, further inform and engage with the team on the ground to realise that plan.

#### Expert networks and relationships

The TNCfMI is a collaboration led by the Royal College of Obstetricians and Gynaecologists and the Royal College of Midwives, which represent the vast majority of UK maternity care professionals. This is a major advantage, as this may foster doctors’ and midwives’ trust in the programme. Additionally, the TNCfMI team comprises a multidisciplinary group of professionals who are known for expertise in their fields, and who foster wide networks. They were approachable and willing to support the early adopter teams, and their professional reputations may have encouraged others to participate in the Community of Practice workshops during the Tool’s early development.

#### Additional dedicated resources

Originally, local leads identified individuals who they felt would be willing and able to take on the role of project champions. However, it became apparent that there was a need to have dedicated paid staff, not just volunteer champions, to plan, manage and oversee the implementation:

*“…in an ideal world if we could do it again, I think you would have like a proper 12 to 24 months of a project officer who would help support that safety netting process.” [ID-HCP017]*

The third maternity service, which launched in July 2022, employed a dedicated admin assistant, supported by short-term funding secured by the maternity service, who followed up newly booked women not already registered on the Tool. This investment in admin time saved midwifery time, reduced clinic waiting times and the chance of assessments being rushed or missed completely.

#### Reinforcement of potential benefits

Many HCP respondents were excited by the Tool and saw its potential for standardising care and reducing workload. It was important, however, that these hopes were realised relatively quickly. One of these benefits was expected to be a reduction in the number of growth scans, as it was expected that more women would be categorised as not requiring them. After several months, following an initial increase, Maternity Service A saw a reduction from around 2 growth scans per pregnancy when the programme was launched in November 2021, to around 1.6 in January 2023. In Maternity Service B the number of growth scans dropped by 8% between 2021–22 and 2022–23. Staff training included reinforcement of potential benefits, a summary of which is shown in Table 5.

**Table 5:** Potential benefits from implementation the Tommy's Clinical Decision Support Tool

The tool aims to improve risk assessment accuracy and decision support for informed decision making. **Potential benefits of implementing the Tommy’s Tool are…**
**for women:**
A more accurate risk assessment and improved decision support aims to ensure that women are offered the right care at the right time, no matter where they live. For some women this will mean avoiding unnecessary appointments and for others this might mean being offered low dose aspirin, additional scans, or referral to a specialist clinic The Tommy’s Tool also offers women: 1. Direct access to their care recommendations, noting the care being offered and when that care should be offered during pregnancy, to ensure they are well informed and able to self-advocate 2. Direct access to its Information Hub which links directly to high quality evidence-based pregnancy information from trusted sources, such as the NHS, the RCOG and Tommy’s charity
**for healthcare professionals:**
A more accurate risk assessment and improved decision support aims to ensure that midwives and doctors: • are more likely to provide appropriate care for the women and pregnant people who need it most • are able to more efficiently direct limited resource by avoiding unnecessary interventions Improved and instantaneous clinical decision support based on national clinical guidance means that midwives and doctors: • can be reassured that the care recommendations offered are based on national clinical guidance • can be re-assured that women and pregnant people have access to their care recommendations in appropriate language to support communication and informed decision making • are supported to standardise care in line with national guidance as a minimum and therefore, should spend less time ‘chasing’ care plans from different consultants. The implementation guidance and training is clear that care recommendations emerging from the Tommy’s Tool risk assessment must be offered as a minimum standard in line with national clinical guidance, and additional care and monitoring can be added according to clinical judgement
**for the Maternity Service**
A more accurate risk assessment and improved decision support aims to ensure that maternity services may: • reduce variation in care within the service and in comparison with other maternity services • free up clinician time in an overstretched workforce, improving staff morale, reducing stress, burn out and sickness • save costs by reducing unnecessary interventions • reduce rates of stillbirth and preterm birth • can demonstrate that they are complying with Saving Babies Lives Care Bundle • free up sonographer slots, so they are available for women who really need them, and the potential to offer more women growth scans at 36 weeks

#### Wider policy initiatives

Implementing a novel intervention that fits well with the wider political policy context is helpful, and reducing stillbirth and preterm birth rates in the UK remains a priority. The Saving Babies’ Lives Care Bundle (SBLCB) [10], sets out evidence-based guidance for maternity services to achieve a UK Government target to halve stillbirths by 2030, and includes guidance on: reducing smoking in pregnancy; risk assessment and surveillance for fetal growth restriction; raising awareness of reduced fetal movement, effective fetal monitoring during labour and care of women at risk of preterm birth. This followed a new UK Government target to reduce the preterm birth rate from 8 to 6% by 2025 [11]. The Tommy’s Tool assessments are in line with SBLCB, which makes the care pathways it recommends familiar and acceptable, and should make achieving the standards easier.

### Fidelity

#### Was the implementation strategy carried out as planned?

There were delays to Tool launch in all maternity services. This was largely due to the COVID-19 pandemic, and its impact on the NHS, but also delays in securing local approvals and staff changes at leadership level. Initially, many women arriving for first appointments were unaware of the need to register on the Tool in advance and some HCPs reported that they did not remember receiving any training, or that it was minimal. These issues were addressed by adaptations, which are detailed, along with their rationale, in Additional file 4.

#### Did intended users register to use it?

The proportion of maternity service users being registered on the Tool increased from 70% to 90% after July 2022, when the functionality allowing HCPs to register on their behalf was introduced (Table 6):

**Table 6:** Number and proportion maternity bookings and pregnant users registered and verified on Tommy's Tool at each maternity service

Maternity service	Oct 2021 to June 2022			July 2022 to May 2023		
	MSDS*	Tool	%	MSDS	Tool	%
A	542	386	71.2	542	510	90.0
B	318	230	72.2	313	285	90.9
C		N/A**		717	623	86.8

^*^Maternity Service A based on estimate of 6.5K bookings per year. Other average bookings based on Maternity Services Dataset (MSDS) ** Maternity Service C launched Tool in July 2022

The remaining 10% may be accounted for by maternity service users with multiple pregnancies (for whom the Tool is not appropriate) and discrepancies between actual booking numbers and those reported on MSDS. Maternity services reported that MSDS data included women receiving some maternity care at their hospitals, but who were booked elsewhere.

#### Did the Tool work as intended?

The majority of maternity service user survey respondents had no problems accessing or registering to use the Tool, understanding the questions about previous pregnancies or medical history or entering the information (Table 7).

**Table 7:** Maternity service user survey respondents first encounter with the Tool

	Maternity service user survey responses							
Did you:	**No**		**Yes**			**Don’t know/** **Can’t remember**	**Total**	***Missing***
Have any problems accessing it?	699	77.1%	144		15.9%	64	907	*262*
Have any problems registering to use it?	807	89.4%	33		3.7%	63	903	*266*
Have any problems understanding questions about previous pregnancies or medical history?	836	93.2%	17		1.9%	44	897	*272*
Have any problems entering this information?	505	90.3%	16		2.9%	38	559	*273*

The Tool also provides links to reliable sources of pregnancy information to support decision making and raise concerns. Survey respondents who had accessed at least one of the Tool’s Information Hub links (n = 671) were asked to complete additional questions about their experience. Responses are shown in Table 8.

**Table 8:** Maternity service user respondents' views and experiences of the Information Hub

If you viewed any of the information pages, please indicate below whether you agree or disagree with the following statements:	Agree (n)	All (n)	Agree (%)
I found the information I was looking for	556	671	82.9
The information I found made me feel reassured	483	670	72.1
The information I found made me feel anxious	84	669	12.6
The information I found was confusing	62	670	9.3
The information I found helped me to decide whether to contact my midwife for more advice	385	669	57.5
The information I found helped me to decide whether to go to the hospital for checks	385	669	57.5
The information I found helped me to raise my concerns with my midwife/doctor	391	668	58.5

The majority (82.9%) found the information they were looking for and said that it was reassuring (72.1%). It also helped over half (57.5%) to decide whether to contact their midwife and go to hospital for further checks. Slightly more (58.5%) also said it helped them to raise concerns with their midwife or doctor.

#### Were the Tool assessments carried out as frequently as intended?

The number of maternity service users registered on the Tool at the end of the study data collection period was 21,132. The first two assessments, for risk of preterm birth and placental dysfunction, were completed for all those eligible, i.e. excluding those with multiple pregnancy or pre-existing hypertension or diabetes. It was not possible to determine how often the changes in fetal movements assessment had been carried out appropriately because data was not available on the actual number of women who had presented with changes in fetal movements. Similarly, data was not available on numbers of women presenting with symptoms of possible preterm labour, but it is very likely, at 1.1%, that it was not used as frequently as expected. This may have been due to a global shortage of fetal fibronectin tests and lack of capacity to provide cervical length ultrasound scans. It may also have been because staff were familiar with using the alternative QUiPP app [12] for assessing risk of preterm birth in women with these symptoms.

#### Were the proportions of women in each risk category as expected?

In the absence of published data, the expected proportions in each risk category were agreed by consensus during the Community of Practice workshops. Data from the TNCfMI QMS reports indicated that the proportions of maternity services users in each risk category were as expected (Table 9).

**Table 9:** Expected vs actual proportions of maternity service users risk categorisation for Tommy’s Tool preterm birth and placental function assessments

		QMS report date		
Tool touchpoint & risk category	Expected*	July 2022	Oct 2022	May 2023
Initial preterm birth assessment				
Moderate	5.0%	4.8%	5.0%	5.7%
High	5.0%	5.6%	5.4%	5.5%
Initial placental function assessment				
Moderate	16.0%	14.2%	14.2%	16.5%
High	8.0%	7.6%	8.0%	7.9%

^*^expected rates agreed at Community of Practice workshops

#### Was data on care pathways and pregnancy outcomes collected as intended?

Completion of the Tool’s pregnancy outcome survey (POS), where minimal outcomes and compliance with care recommendations are documented, was low, at only 12.1% (2,562/21,132) of registered pregnancies. Sites reported that this was due to lack of staff capacity, and the burden of inputting data that had already been entered on the main record system with no perceived benefit from duplicate of effort. Hospitals were encouraged to prioritise records of women experiencing poor outcomes, so rates of these outcomes could be monitored. The Tool was also adapted to enable notifications to users if the POS remained incomplete six weeks after the expected date of delivery.

#### Were care pathways followed?

It was not possible to evaluate how often the recommended care pathways were followed because this depended on completion of the Tool’s pregnancy outcome survey, which had not been completed in all cases.

#### Can we rely on maternity service users' reports to evaluate fidelity?

We asked maternity service user survey respondents what the results of their risk assessments were. Of all the respondents who answered this question (n = 1,058), 42.2% could not remember either what the preterm birth risk assessment result was, or that the assessment had been carried out at all. Exactly the same proportion (42.2% of 1,033) of women could not remember the placental function assessment result or whether the placental function assessment had been carried out. This led us to conclude that self-report is not a reliable method for collecting data to evaluate fidelity.

### Unintended consequences

#### Impact of unexpected risk assessment results

The Tool’s algorithms are more likely to correctly identify high risk women and de-escalate risk and reduce intervention for others. However, this can lead to unexpectedly high-risk results that could cause anxiety, compounded if the HCP was unable to explain why, or how the Tool had come to its conclusion:

*“… when she said I was high risk I came out and I cried… I was totally in denial that I should be at high risk, because I couldn't... I don't think it was fully explained to me the reasons why, and I do wish I knew the reasons as to why I did come out as high risk.” [ID-WM022]*

Most women assessed as low risk found it reassuring. Again, however, unexpected low risk results, e.g., for women who had previously experienced complications, could also lead to uncertainty and anxiety, if they were told they did not need the additional scans and monitoring they expected.

For some women, a high-risk assessment which includes current clinical test results could be more concerning than assessments based only on a checklist. This may, however, increase motivation to comply with recommended care, such as taking daily aspirin [13].

Communication support to HCPs during appointments was improved through the addition of a ‘switch to woman and birthing person view’ function on the HCP user interface. This feature enables the HCP to display the care pathway overview as it appears on the woman’s user profile, thereby communication can focus on care recommendations offered and why those care recommendations are offered in language suitable to the lay audience, rather than leading with what can be a jarring presentation of a red, amber, green description of risk commonly used by HCPs. This feature was developed with valuable input from a dedicated advisory group of women with lived experience.

For example *'in addition to routine antenatal care you will be offered additional scans at x and y weeks’ gestation. These additional scans are offered so we can monitor your baby’s growth during pregnancy.*’

#### Concerns about risk results and care recommendations

Twenty-six HCP survey respondents (35.6%, 26/73) indicated they had been concerned about the result of a risk assessment on at least one occasion, while 18 (25.0%, 18/72) had had concern about a recommended care pathway. Some said they felt the Tool was too simplistic while people and pregnancies are complicated. Others were concerned that the use of algorithms could make results difficult to explain, when the reason for the result was not obvious. Some women believed that HCPs were compelled to follow the care recommendations and were concerned that the Tool was overriding clinicians’ judgements, which they were not happy about. These concerns and misunderstandings were allayed by the information included in the implementation toolkit and training resources as well as the information for women and pregnant people which states that care recommendations emerging from each risk assessment are offered as a minimum standard in line with national clinical guidance, and that care can always be escalated based on clinical judgement but must never be de-escalated. Recognising the importance of this message, this was further addressed in a revised training package to establish this principle.

## Adaptations

The need to understand local context and ability to adapt is a fundamental principle of implementation [9]. Greenhalgh and colleagues’ NASSS framework shows how the process of implementation requires constant review and adaptation. During the early adopter implementation phase, direct feedback from staff and women, combined with findings from this study, were used to support sites who needed to change practices to bring them in alignment with the Tool. We also used this feedback to inform adaptations to the Tool itself, and to the implementation and training strategies.

Tommy’s Tool assessments incorporate several test results that are not currently universal within UK maternity services. When necessary, the TNCfMI team worked with local staff to find resolutions and adapt procedures and practices. The issues identified that could impact on successful implementation, along with the resolution, are described in detail in Additional file 2: “Adaptations to local practice required during implementation of Tommy’s Tool”.

A series of adaptations were made to the Tool which included changes to wording, to clarify meanings and reduce errors, as well as functionality, e.g. to allow HCP users to register details on behalf of women, and to 'switch to ‘women and pregnant people view’ of the care pathway overview, alerts to remind users to complete pregnancy outcome details and the introduction of a training platform. These are itemised, along with the rationale for the changes, in Additional file 3: “Adaptations to Tool during Early Adopter Implementation”. Successful implementation of a novel complex intervention also requires consideration of, and adaptation to, local context. Adaptations to the implementation and training strategies, along with their rationale, are described in Additional file 4: “Adaptations to Implementation and training strategies during early adopter implementation”.

Future adaptations planned include translation of the maternity service user interface into common non-English languages, to increase usability for non-English speakers. Plans also include integration of the Tool with maternity information systems to reduce the burden of double data entry. These adaptations are planned for after completion of the RCT because, apart from minor bug fixes and resolution of safety issues, changes to the intervention cannot occur during the trial period.

## Discussion

### Key learnings from the early adopter implementation

One of our key learnings was the importance of *clarity on the purpose and scope* of the intervention. It is a clinical decision support tool, and changing its name, from the Tommy’s App, was an important move towards clarifying its scope and purpose. The functionality allowing HCPs to register women who were unable or unwilling to engage themselves is arguably the most important of all the adaptations made. After this change, the proportion of maternity service users registered on the Tool rose from around 70% of those booked for maternity care, to around 90%, as shown in Table 6.

*Emphasising the potential benefits* of the Tool was also important, as outlined above. Whether the Tool will influence other care, e.g. rates of induction of labour and caesarean section, will be determined in the RCT. After the introduction of SBLCB [14] rates of these interventions appeared to rise [15].

That the Tommy’s Tool *fits within wider policy initiatives* makes successful implementation more likely, as hospitals need to demonstrate they are complying with national guidance to benefit from the NHS’s Maternity Incentive Scheme. The UK’s National Health Service (NHS) Long Term Plan [16] aims to ensure national programmes are focused on reducing health inequalities and addressing unwarranted variation in care, whilst empowering people to have more control over their own health, and more personalised care when they need it. We know that women from underserved groups—those from ethnic minority backgrounds and/or areas of the greatest social deprivation—are more likely to experience poor pregnancy outcomes [17, 18], and it is hoped that the Tool will reduce inequity in care and outcomes. In their retrospective cohort study, carried out in a large South London hospital [19]. Liu and colleagues compared outcomes of women screened using NICE criteria with the Fetal Medicine Foundation algorithm, which is used in the Tool’s placental function assessment. They found not only a reduction in poor outcomes, but a levelling of the rate of perinatal death between white and non-white women who had been risk assessed using the FMF algorithm.

Stakeholder involvement is a key factor in successful implementation, so *multidisciplinary “buy-in” from the start* is essential. Whilst development of the Tool involved experts from a variety of professional disciplines and maternity service users, implementation in the maternity services tended to be led, at least initially, by senior obstetricians, with less involvement from midwives and sonographers. The wider and more diverse the teams grew, the smoother the implementation became.

Implementation of system-wide novel interventions requires *preparation for a transitional period.* One of the most significant issues raised by Tommy’s Tool leaders and champions was the danger of providing different care pathways simultaneously. Harmonisation of guidelines in advance of launching the new intervention may help to ease this transition. Some adaptations to practices can be put in place for all women booking for maternity care, in advance. In the case of Tommy’s Tool, this could mean providing PAPP-A results for all, introducing standardised blood pressure measurements and moving uterine artery Doppler screening to the first trimester scan.

It is very difficult to implement novel interventions or practices without *dedicated resources*. In additional to local volunteer champions, it became clear that a dedicated senior member of staff should be appointed to plan and manage the practical implementation as part of their paid role. As midwives are the largest professional group, this would preferably be a midwife (e.g. Maternity Transformation Lead, Digital or Saving Babies Lives Midwife). It is also very important that the team working with the Lead, from the outset, is multidisciplinary, including midwives and sonographers, as well as obstetricians and neonatologists. Contingency plans should be in place to allow rapid and smooth transition if leads and champions leave their posts. Additionally, dedicated administrative support could be utilised, e.g. to monitor registrations and follow up when women have not yet registered before booking.

Digital interventions require *optimisation of the hospital’s IT infrastructure*. Digital maturity in NHS maternity services was identified as being suboptimal in 2018 [20], and readiness for digitisation in hospitals varies widely [21]. Sites implementing the Tommy’s Tool in future will need to have a sufficient level of digital maturity to enable successful implementation. Mitigation of double-data entry to reduce burden for staff remains a priority, and although there are plans to integrate the Tool with maternity information systems, this undertaking will require significant policy level influence, resource and time to achieve.

Ensuring *flexibility and accessibility in training and resource materials* is key. Initially, training was undertaken locally with resources provided by the TNCfMI team. However, a significant proportion of HCP participants reported receiving little or no initial training. Some also reported having difficulties finding the resources as they were stored behind hospital firewalls, which were sometimes difficult to access, particularly by those working in the community. Adjustments to the training strategy were made, including plans to ensure staff received training at the right time, i.e. not too long before they needed to apply the learning. Local champions were supported in training others (cascade training), online “drop-in” sessions were established and the toolkit was expanded to include a “Frequently Asked Questions” document offering comprehensive informed responses. A new web-based training platform was also released, identical to the live application with additional pre-set scenarios and a revised Implementation Toolkit will also be available on the internet.

*Continuous embedding and adaptation over time* is an important factor in successful implementation, as described in Domain 7 of the NASSS framework [9]. The difficulties of sustainability and successful scale up depend largely on how well a novel intervention or practice can be adapted. In this early adopter study, we were able to make adaptations to the Tool and implementation strategy, which appeared to make implementation in Maternity Service C smoother, but there is still more to learn. An implementation evaluation work stream will run alongside the RCT’s main trial and cost effectiveness workstreams.

This early adopter implementation relied on the availability of additional resources, as well as goodwill and good relationships. As the programme moves forward, with the RCT and ultimately wider scale up in the NHS, it will not be possible for the TNCfMI to maintain the level of support they were able to during the early adopter phase. This underlines the importance of early adopter implementation evaluation which is designed to optimise the intervention and implementation strategies and develop an ongoing, albeit scaled-down, support strategy.

In the longer term, as a digital technology, the Tool can be adapted relatively quickly and easily, as new evidence and guidelines become available, which increases its chances of sustainability over time.

### Implications for the forthcoming RCT

What we have learned will not only increase the chance of successful implementation in the trial sites but also improve the quality of the RCT data. In this early adopter implementation study, the available data was not sufficient to reliably evaluate fidelity of individual Tool assessments. This was due to incompleteness of the Tool’s pregnancy outcome survey and the unreliable nature of self-reported and MSDS data. In the cluster RCT, we will use data from the hospital health record systems and triangulate it with data collected through the Tool to ensure completeness. Evaluating fidelity is a vital component of intervention trials, particularly cluster RCTs of complex interventions. If there is little or no difference in the primary outcome, it may be because: a) the intervention is not being used as it should, or b) the practices are similar in the control sites. Researchers carrying out the Design trial [22], which evaluated the implementation of a complex intervention to improve detection of small for gestational age (SGA) babies, found no difference in the primary outcome. This may have been due to variable implementation fidelity in the intervention sites, and implementation of some of the elements of Saving Babies Lives Care Bundle designed to reduce SGA in control sites.

We have also refined the recruitment strategy, allowing face-to-face approach of prospective interview participants, as well as email and text messaging, to widen the diversity of participants. We have also revised our online questionnaires, making them shorter and less burdensome.

### Strengths and limitations

This study utilised a multi-methods approach in order to gather as much data as possible from a variety of sources. However, self-selection, an inevitable consequence of a consenting cohort study, will result in bias. Maternity service users who participated in this study were a small proportion of all those registering on the Tool. Others may have had different views from those choosing to participate. Despite purposive sampling, fewer women from ethnic minority groups accepted our invitation to interviews or focus groups, and there were no respondents from non-English speakers or under 16-year-olds. Evaluation of fidelity was limited due to poor completion of the Tool’s pregnancy outcome survey.

## Conclusion

This study evaluated the implementation of the Tommy’s Clinical Decision Support Tool in four NHS maternity services ahead of a forthcoming RCT where intervention efficacy and cost consequences will be evaluated. Despite delays due to the COVID-19 pandemic, we were able to successfully implement the Tool in three of the four intended NHS Trusts. In a context where staff so recently had to deal with major challenges and changes in practice, it is remarkable that the first maternity services were able to launch at all. This was largely due to the commitment and determination of the local leads and champions, with ongoing support from the TNCfMI team. In wider scale up, this outside support would not be sustainable, which is why optimisation at this early stage was crucial. This implementation study gave us the opportunity to optimise the Tool and implementation strategy, as well as refining the RCT protocol, which will increase the chances of the trial findings being meaningful and conclusive.

## Supplementary Information

Additional file 1: “Checklist” Checklist to guide pre-launch preparations

Addtional file 2: “Adaptations to local practice required during implementation of Tommy’s Tool” List of adaptations to local practice with rationale for adaptation and resolution

Additional file 3: “Adaptations to Tool during Early Adopter Implementation” List of adaptations to Tommy’s Tool with rationale for adaptation

Additional file 4: “Adaptations to Implementation and training strategies during early adopter implementation” List of adaptations to implementation and training strategies with rationale for adaptation

## Data Availability

The qualitative datasets generated during the current study are not publicly available due small numbers making it impossible to adequately anonymise the data. Online survey participants did not consent to the sharing of data for other research purposes.

## Abbreviations

ANQ: Antenatal questionnaire
FMF: Fetal Medicine Foundation
HCP: Healthcare professional/provider
HRA: Health Research Authority
ID-HCP##: Identifier for health professional interview or focus group participant
ID-WM##: Identifier for maternity service user interview or focus group participant
IMD: Index of multiple deprivation
ISRCTN: International Standard Randomised Controlled Trial Number
IT: Information technology
MHRA: Medicines and Healthcare Products Regulatory Agency
MSDS: Maternity Services Dataset
NASSS: Non-adoption or Abandonment of technology by individuals and difficulties achieving Scale-up, Spread and Sustainability
NHS: National Health Service
NICE: National Institute for Health and Care Excellence
NIHR: National Institute for Health Research
PAPP-A: Pregnancy Associated Plasma Protein-A
POS: Pregnancy outcome survey
QMS: Quality Management System
RCOG: Royal College of Obstetricians and Gynaecologists
RCT: Randomised controlled trial
REC: Research Ethics Committee
SBLCB: Saving Babies Lives Care Bundle
SGA: Small for gestational age
TNCfMI: Tommy’s National Centre for Maternity Improvement
UK: United Kingdom
