# Supplementary material for: Tommy’s Clinical Decision Support Tool: an intervention development and feasibility study to inform a future randomised controlled trial
Source: Pilot Feasibility Stud. 2026 Feb 25;12:45. doi: 10.1186/s40814-026-01788-9 (PMC13041263; doi:10.1186/s40814-026-01788-9)
Supplement: Supplementary file 1 — Additional file 1: “Checklist” Checklist to guide pre-launch preparations [file 40814_2026_1788_MOESM1_ESM.pdf]

		IMPLEMENTATION CRITERIA						
		Requirement	Desirable / Essential	Responsibility	Not In Place	In Progress	In Place	Notes [examples given below]
1	CLINICAL GOVERNANCE							
	1	Register as Trust Quality Improvement (QI/Service Improvement Project)	Essential	Local site				1. UPDATE JUNE: Project is not registered under QI but funding can be requested as and when required<br>2. UPDATE JULY: Implement first as an innovation project - then evaluate improvement needs - complete
	1	Approval by site Clinical governance or Management team for implementation (CD, HOM, DOM sign off site agreement)	Essential	Local site				1. UPDATE JUNE: Tommy's team to issue final letter to Clinical Director with appendices<br>2. UPDATE AUGUST: Letter to be sent to the Clinical Director [name] W/C [date] - complete
2	RESEARCH GOVERNANCE							
	2	Approval by site R&D department for improvement science evaluation of Tool	Desirable	Local site				1. UPDATE JUNE: Sponsor comments on the Evaulation Study protocol currently being reviewed by [name]<br>2. UPDATE AUGUST: REC meeting to be held on [date] and CRN support confirmed. Local Research Midwives are [name] and [name]<br>3. UPDATE JAN 2022: WITH GO LIVE DATE NOW IN PLACE, ACTION [name] TO PICK UP WITH R&D
	2	Nominate PI for evaluation study	Essential	Local site				1. UPDATE APRIL: [name] (PI) and [name] (Obs Lead) confirmed locally<br>2. UPDATE DECEMBER: [name] to take over from [name] as the PI
3	IMPLEMENTATION							
	3	Trust received Tommy's Clinical Decision Tool implementation toolkit	Essential	Tommy's				1. UPDATE MARCH: Batch 1 of Implementation Toolkit materials sent ([date])<br>2. UPDATE MAY: overview and pathway run through from Tommy's Team and batch 2 materials sent ([date])<br>3. UPDATE JUNE: Final issue Implementation toolkit and resources will be shared to follow June meeting<br>4. UPDATE JULY: final toolkit issue plus HCP presentation sent [date]<br>5. UPDATE JULY: ACTION: [name] to resend toolkit to [name] - complete<br>6. UPDATE AUGUST: [name] and [name] to ensure local Toolkit version control (Tommy's to inform local team of any updates)<br>7. UPDATE JAN 22: [name] TO TAKE ON MANAGEMENT OF TOOLKIT LOCALLY
	3	Site champions, including women's representation, appointed and have time to support initial implementation	Essential	Local site				1. UPDATE MAY - Named site champions required: Obs, MW (community based/hospital based), Digital Midwife (if applicable), Sonographer, local MVP lead and Maternity IT<br>2. UPDATE JUNE: ACTION: [name] and [name] to confirm site leads either at or before July site meeting<br>3. UPDATE AUGUST: ACTION: [name] and [name] to confirm site champions (Obs, MW (community based/hospital based), Sonographer/Labour Ward) at or before September site meeting<br>4. UPDATE OCTOBER - [name] and [name] to confirm<br>5. UPDATE DECEMBER: Confirmed that the Community Team Leaders, DAU Team Leader and [name] will be the site champions

	IMPLEMENTATION CRITERIA						
	Requirement	Desirable / Essential	Responsibility	Not In Place	In Progress	In Place	Notes [examples given below]
3	All Trust guidelines aligned with Clinical Decision Tool	Desirable	Local site				1. UPDATE JUNE: [name] and [name] to advise on any support required from the Tommy's team to progress this<br>2. UPDATE JULY: ACTION: Tommy's team to share guideline cover sheet examples from other sites<br>3. UPDATE AUGUST: [name] and [name] to progress locally<br>4. UPDATE OCTOBER: final stage of sign off mid October ([name] and [name] managing this)<br>5. UPDATE DECEMBER: Coversheets have been signed off - to be added to the guidelines<br>6. UPDATE JAN 22: Signed off and to be implemented on local intranet
3	Training of all maternity staff in use of Tool and specifically to ensure:	Essential	Local site				1. UPDATE JUNE: Test and Learn platform now available and leads will be added as HCP users<br>ACTION: [Tommy's Centre Lead] and team to attend Grade 7 meeting to present app concept and benefits<br>2. UPDATE JULY: Tommy's to meet with the education team on [date] to plan for local training and to review the toolkit - complete<br>ACTION: [local champions names] to send maternity manager's email addresses to Tommy's<br>3. UPDATE AUGUST: ACTION: [Local Obs Lead] to lead the Obs trainee induction<br>ACTION:Tommy's to arrange local access to the staging platform<br>ACTION: [MW Lead] to send [the Community Midwives team leaders email addresses for access to the staging platform - complete 9.<br>ACTION: [PI name] to send [admin] the email addresses for labour ward staff for access to the staging platform - complete<br>4. UPDATE OCTOBER: [champion name] leading on plan for training with the support of the Education team. Reconfiguration of Community team at present.<br>5. UPDATE DECEMBER: ACTION: Meeting with [local champion names, Tommy's Practical Implementation Midwife] to be arranged to discuss the training plan and shared learning from the other early adopter sites - [champion name] to suggest dates (intended Jan start)<br>6. UPDATE JAN 22: MIDWIFERY TRAINING PAUSED AT PRESENT BUT REVIEW THIS WEEK AS TO WHETHER CAN RECOMMENCE (LOOKING AT POSSIBILITY OF REMOTE TRAINING START AT END OF JAN
	3.4.1 Community midwives prepared/given supporting information for booking assessments and other key touch points	Essential	Local site				
	3.4.2 Awareness of information facility for pregnancy concerns and 'synergy' in advice given by staff	Essential	Local site				
	3.4.3 Support information for women	Essential	Local site				

	IMPLEMENTATION CRITERIA						
	Requirement	Desirable / Essential	Responsibility	Not In Place	In Progress	In Place	Notes [examples given below]
4	Process in place to send out invitation letter/QR code to maternity service user with booking appointment	Essential	Tommy's				1. UPDATE JUNE: No letters are sent. The app download link to be included in booking text message and in the self-referral screening information sent to women<br>2. UPDATE JULY: [Tommy's Centre team member name] to pick up with [name] and IT team at Trust - UPDATE AUGUST: ACTION: [name] to contact [name] today<br>4. UPDATE AUGUST: suggest adding app information to the Trust self-referral webpage - ACTION: [name] to discuss with [name]<br>5. UPDATE SEPTEMBER: ACTION: [name] to liaise with [name] about sending women a link to register for the app in the email confirming self-referral<br>6. UPDATE OCTOBER: Booking info is sent via automated text - action to include hyperlink to the Invitation letter and patient information which should be uploaded to the Trust website<br>NOTE: [new champion name] has taken over from [champion name]<br>7. UPDATE DECEMBER: local arrangements are underway to send women an email containing info on the app.
4	Process in place for completion of birth details on Tommy's Clinical Decision Tool	Essential	Local site				1. UPDATE FEB: birth midwife/discharging co-ordinator (yet to be decided)<br>2. UPDATE JUNE: Site confirmed birthing Midwife on the Labour Ward to complete birth details<br>3. UPDATE AUGUST: Suggested a local checklist is created for staff to complete the birth details within the app<br>4. UPDATE AUGUST: Local process to note miscarriages within the app to be discussed - to be discussed at training planning meeting<br>5. UPDATE SEPTEMBER: Intrapartum midwife to complete birth details ([PI name] confirmed)<br>6. UPDATE OCTOBER: review this as part of the whole end to end local process once established (in the interim share site 1 and 2 process for this)<br>7. UPDATE DECEMBER: Intrapartum Midwife would complete the birth details<br>8. UPDATE JAN 22: STANDARD BOOKING INFO SHARED ONLINE TO BE POPULATED WITH RELEVANT PATIENT FACING INFORMATION FROM IMPLEMENTATION TOOLKIT
4	IT SUPPORT						
4	Process in place to access Tommy's Clinical Decision Tool on Trust IT systems	Essential	Local site				1. UPDATE MAY: Informatics Midwife [name] meeting with [Tommy's Centre team member] - complete<br>2. UPDATE JUNE: IT brief, security info and DPIA to be shared as appendice to Governance letter - [name] is aware<br>3. UPDATE SEPTEMBER: ACTION: [champion name] to seek an update from [name] on trust IT progress and will update Tommy's team<br>4. UPDATE OCTOBER: [Tommy's Centre team member] to pick up with [champion name] and local IT<br>5. DECEMBER UPDATE: ACTION: [Tommy's Centre team member] to pick up with [Tommy's Centre team member] re IT update next week
5	PRETERM BIRTH SERVICES						

	IMPLEMENTATION CRITERIA						
	Requirement	Desirable / Essential	Responsibility	Not In Place	In Progress	In Place	Notes [examples given below]
5	Process in place to perform preterm birth assessment in first trimester	Essential	Local site				1. UPDATE JUNE: ACTION: [Local preterm lead name] to draft HCP and patient facing infographic on pre-term birth assessment and submit to Tommy's Centre Team for review - complete
5	Process in place to perform mid-trimester Cx length in moderate risk women	Essential	Local site				
5	Process in place to perform possible preterm labour assessment – Out of Hours Cx Length	Desirable	Local site				Not routine; (5.3 and 5.4 - 1 of 2 is essential)
5	Process in place to perform possible preterm labour assessment – Fibronectin	Desirable	Local site				1. UPDATE MARCH: All relevant staff are trained.
6	ULTRASONOGRAPHY SERVICES						
6	Process in place to perform placental function assessment - PAPP-A for all women	Essential	Local site				1. UPDATE MARCH: Recommendation that this be introduced to all women - not yet in place<br>2. UPDATE JULY: Process in discussion locally (10-13+6 /UP TO crl 84MM)<br>3. UPDATE AUGUST: Guideline is progressing locally<br>4. UPDATE ON THIS BY EMAIL TO FOLLOW MEETING<br>5. UPDATE DECEMBER: Booking Midwife to discuss PAPP-A with women<br>6. UPDATE DECEMBER: The community midwife to input the PAPP-A and uterine artery Doppler results at 16 weeks<br>7. UPDATE JAN 22: COMMS TO SONOGS REQUIRED TO CONFIRM PROCESS FOR WOMEN WHO DECLINE SCREENING BUT STILL HAVE PAPP-A
6	Process in place to perform placental function assessment - 1st trim uterine artery Doppler (Note: Not essential - but will dramatically increase accuracy)	Desirable	Local site				1.UPDATE MAY: Meeting with Sonogs team<br>2. UPDATE JUNE: In progress and expected to be in place by switch on in September<br>3. UPDATE JULY: On track for UtAD<br>4. UPDATE AUGUST: ACTION: [local Obs lead name] to seek an update on UtAD training progress<br>5. UPDATE SEPTEMBER: Training at [site name] is progressing and update to be sought from [USS lead name]
6	Process in place to perform placental function assessment - System for prescribing Aspirin	Essential	Local site				1. UPDATE MARCH: PGD paperwork sent by Tommy's. In progress at site ([name])<br>2. UPDATE JUNE: [name] progressing PGD locally<br>3. UPDATE JULY: Ongoing discussion with pharmacy.<br>4. UPDATE SEPTEMBER: ACTION: [name] to follow up with local pharmacist on progress<br>5. UPDATE OCTOBER: PGD not required - confirmed with [name] and [name]<br>6. UPDATE JAN 22: [name] to update on Aspirin prescription process and [Tommy's Centre Practical Implementation Midwife] on standby to speak with pharmacist if required

	Requirement	Desirable / Essential	Responsibility	Not In Place	In Progress	In Place	Notes [examples given below]
	IMPLEMENTATION CRITERIA						
6	Process in place for the timing of birth assessment - USS incl MCA Doppler	Desirable	Local site				1. UPDATE JUNE: MCA doppler training is partially rolled out and training is planned/in progress for the remainder. i.e. Rolling out after the 1st trim uterine artery Doppler training is in place<br>2. UPDATE JUNE: ACTION: [name] to arrange a 1hr meeting with [Tommy's Centre Clinical Lead] and sonographers - ideally on the same day as the Grade 7 meeting (also noted as an action on item 3.4 above) - outstanding<br>3. UPDATE JULY: ACTION: [name] video to be shared to assist - outstanding - video to be shared with the site<br>4. UPDATE JAN 22: SOME TRAINING OUTSTANDING ([name] TO LEAD) TO ENSURE COMPLETE AND MCA PERFORMED CONSISTENTLY ACROSS THE TEAM
7	DAY/MATERNITY ASSESSMENT SERVICES						
7	Process in place to perform change in FM assessment - cCTG	Essential	Local site				
7	Site initiation meeting	Essential	Tommy's				1. UPDATE JUNE: Suggest end of August meeting for site initiation at [hospital name]<br>2. UPDATE AUGUST: Suggest end of September for site initiation at [hospital name]<br>3. UPDATE OCTOBER: Switch on planned for #th December - HCP set up required two/three weeks prior<br>4. UPDATE DECEMBER: Switch on planned for ##/##/2022
