# Supplementary material for: Tommy’s Clinical Decision Support Tool: an intervention development and feasibility study to inform a future randomised controlled trial
Source: Pilot Feasibility Stud. 2026 Feb 25;12:45. doi: 10.1186/s40814-026-01788-9 (PMC13041263; doi:10.1186/s40814-026-01788-9)
Supplement: Supplementary file 2 — Addtional file 2: “Adaptations to local practice required during implementation of Tommy’s Tool” List of adaptations to local practice with rationale for adaptation and resolution [file 40814_2026_1788_MOESM2_ESM.pdf]

Additional file 1: Adaptations to local practice required during implementation of Tommy's Tool

Factor	Established practice (in one or more Trust)	Change required for Tommy's Tool	Issues arising	Resolution
PF-A timeframe	Follow up midwife appointment from 16 weeks	PF-A must be run before 16 weeks.	If recommended, aspirin must be commenced before 16 weeks for optimum benefit.	Alignment of appointment schedules to correspond with PF-A timeframe.
PAPP-A blood test	PAPP-A only as part of anomaly screening.	PAPP-A for all women.	Women declining anomaly screening may not have this test.<br>If they do, they may accidentally be given anomaly screening results.	Consent process changed to highlight reasons for test.<br><br>All consenting women have PAPP-A blood test.<br><br>New blood test request form.<br>Sticker on notes/blood form to highlight if only for PFA.<br><br>Communication with HCPs to clarify PAPP-A not mandatory to run PFA, but that it's inclusion increases accuracy of the results.
	Screening midwives contact women when screened positive for fetal anomaly.	Community midwives need to look up and enter result for all women before running PF-A.<br><br>Some midwives not able to access results in the community.	Additional time/burden on CMWs.<br><br>Missing data reduces accuracy of result.	Facility to save clinical test results in advance of PF-A added to Tool functionality.<br><br>Trust C engaging screening midwives to review and enter all PAPP-A results so available when

				CMWs ready to run PF-A.
Blood pressure measurement	One reading at booking	Four readings at booking using validated machine.	BP machines may not be validated.	List of validated machines supplied to Trust. Local champion/site lead checks and orders equipment if necessary.
			Additional time required.	Guideline amended to allow one reading if ≤110/70.<br><br>Reminder to staff that standardised BP likely to result in less women being identified as high risk, thus reducing unnecessary intervention and burden.<br><br>In light of new evidence, number of BP readings required reduced to 3..
Uterine Artery Doppler scan	Performed at 20 weeks' anomaly scan on women at high risk of placenta dysfunction.	Perform at first trimester scan for all women.	Sonographers consenting women who are registered on Tommy's – prolonging appointment. Some women missing out if not yet registered.	UADs performed on all women, whether or not registered. As a routine procedure, no specific consent required.
			Additional time needed during scan appointment	Appointment times extended. Savings made later if fewer growth scans required.
Aspirin prescription	Prescribed to high risk women at	Prescribed by midwife following PFA at	Midwives require PGD to provide aspirin.	Leads pursued implementation of PGD and liaise

	12/40 by doctor in antenatal clinic.	<16/40 appointment.	Pharmacy authorisation required.	with Pharmacy for approval.<br><br>Learning shared between pharmacies at early adopter sites.<br><br>RCOG Aspirin in pregnancy patient information sheet distributed to midwives to support implementation of this new practice.
			Midwives need to carry aspirin supply or identify safe storage spaces in clinics	Central storage with community team, each midwives carry a couple of boxes, replenish as required.
PTB-A	Using checklist approach to risk assessment	Algorithm leads to more women being correctly identified as moderate or high risk	Over stretched preterm service exceeds capacity.	Referrals triaged by preterm teams (some only require USS for cervical length (CL) and preterm clinic appointment if cervix is short).<br><br>Triage flow chart developed by Trust A and shared with others.
			Increase number of CL scans at anomaly scan	No problems reported.
		Midwives unsure about which cervical procedures require referral	Increase burden unnecessarily	Info sheet about different cervical procedures developed by Trust C and shared with others.
PPTL-A	Fetal fibronectin (fFN) and/or CL not	PPTL-A requires either fFN or USS for CL measurement.	Cannot run PPTL-A without one or other test.	Sites encouraged to use fFN and/or CL as part of all PPTL assessments.

	always carried out as part of usual care.			
	National shortage of fFN test kits.			Sites encouraged to increase training in CL, and arrange scans as soon as slot available.
	QUiPP app	PPTL-A uses QUiPP algorithms plus provides care recommendation	If Tool not used, risk and care pathway not recorded on Tool.	Training highlighted to emphasise benefit of using Tool.
CFM-A	Follow SBLCB guidance	No change of practice required as CFM-A based on SBLCB.		
ToB-A	USS for umbilical artery doppler (UmbAD) and fetal middle cerebral arterial (MCA) Doppler not always carried out as part of growth scan.	Addition of these elements increases accuracy of assessment.	ToB-A result less accurate.	Sites encouraged increase training and offer these additional elements.
