# Supplementary material for: Tommy’s Clinical Decision Support Tool: an intervention development and feasibility study to inform a future randomised controlled trial
Source: Pilot Feasibility Stud. 2026 Feb 25;12:45. doi: 10.1186/s40814-026-01788-9 (PMC13041263; doi:10.1186/s40814-026-01788-9)
Supplement: Supplementary file 3 — Additional file 3: “Adaptations to Tool during Early Adopter Implementation” List of adaptations to Tommy’s Tool with rationale for adaptation [file 40814_2026_1788_MOESM3_ESM.pdf]

Additional file 2: Adaptations to Tool during Early Adopter Implementation

Tool version	Date	Adaptations*	Rationale
1.0	Aug 2021	First version of application, hosted by RCOG	
1.1	Dec 2021	Landing page and pregnant user interface: privacy notice update to note move to NHS Digital Hosting.	Device now hosted on NHS Digital Cloud Centre for Excellence.
		HCP interface: CFM-A to include PV bleed/uterine activity.	To ensure these additional factors are taken into account.
1.2	Jan 2022	System admin: data export functionality initiated.	Required for post-market surveillance reports.
		HCP and pregnant user interfaces: wording updates	In response to user feedback.
1.3	Mar 2022	HCP interface: wording change.	To improve clarity of ToB-A recommendation.
		HCP and pregnant user interfaces: mandatory fields for completion appear in highlight until complete.	To improve clarity and data completeness.
		HCP interface: weight limits applied on pregnancy outcome survey fields.	To mitigate erroneous data entry.
		HCP interface: automatic round up applied to CRL value in placental function.	To improve clarity.
		Pregnant user interface: free text box added to medical /obstetric history.	To enable pregnant users to add additional information they would like to share with HCP.
1.4	Jul 2022	HCP interface: functionality to enable HCP to create a pregnant user account on behalf of the pregnant user.	To ensure the care of maternity service users who are unable or unwilling to engage is based on Tool assessments and care recommendations.
		HCP interface: additional functionality to allow PF-A inputs to be 'saved', i.e. 'save draft and run later' or 'save and run now'.	To enable HCPs to add inputs as they become available.
		HCP interface: wording update.	In response to user feedback.
		Pregnant user interface: 'consent to research' wording updated.	To note additional method of contact.
		Landing page and pregnant user interface: privacy notice updated	To improve clarity
		Application of concertina formatting to header sections.	To improve user experience

		Pregnant user interface: rule applied to allow only participating sites to be visible on hospital drop down menu selection.	To avoid inadvertent errors.
1.5	Nov 2022	Local admin user level added.	To allow local management of users, reducing reliance on Tommy's Centre Team.
		Pregnant user interface: change to permissions in adding/ changing/removing a named hospital.	Ensures audit trail of record of care and reason a maternity unit has been removed.
		Functionality offering pregnant users access to her own profile, where original profile was set up on her behalf by HCP.	To enable a second opportunity to encourage pregnant women to engage with the device.
		PF-A: addition of history of stillbirth and/or previous SGA baby will escalate care from moderate to high risk.	In response to feedback from HCPs and women, to escalate care for assurance and additional support. Previously if the woman was a late booker, the application would classify her as a moderate chance placental function assessment, in line with clinically validated algorithm, even if she had a history of stillbirth and/or SGA.
		Functionality to enable an earlier assessment of EDD using LMP.	Enables HCPs to have an earlier estimate of gestation prior to PF-A.
		Additional tab added to ensure pregnancy outcome details are visible to HCP Users and Pregnant Users after the pregnancy outcome details have been completed and saved.	This change ensures that pregnancy history remains readily visible to HCPs for ongoing or future care and for women users as a record of their care that they can own and access whenever they wish to.
1.6	Mar 2023	Additional icon added to HCP user interface that enables the HCP to switch between the HCP and pregnant user view of assessment results.	Enables HCP users to discuss results and care recommendations using pregnant user view. This avoids HCPs sharing their own HCP user view which has a medical format and language.
		Email notification inviting pregnant user to complete POS if empty six weeks after EDD.	A mechanism to ensure the POS is completed.
		Pregnancy outcome details must be verified before submitted and are available to view after completion of POS.	To enable HCP users to view all inputs and details of a pregnancy pathway after the POS is submitted. To enable pregnant users to view previous pregnancy details
		When an HCP user adds a pregnant user’s email address, an email invites them to verify account and set a password for own access.	To make it easier for pregnant user to access account set up by an HCP.

		Language updates	In response to user feedback.
1.7	Sep 2023	HCPs can change the date of LMP and re-run the PTB-A (if the PF-A has not yet been run).	Enables the LMP field to be updated by HCP users. HCP users can return to the medical history, update the LMP and rerun the PTB Assessment.
		HCPs can re-run ToB-A at any time after 36 weeks' gestation. Appropriate warnings will alert them to important information.	Enables the ToB assessment to be re-run by HCP users.
		In-device displays of usernames updated: HCP User and Local Admin User.	For clarity and consistency.
		PTB-A high risk care recommendation wording. 'Your midwife will discuss with you whether you will be offered an appointment with the preterm birth service'.	Previous wording suggested an appointment was always necessary. This is to avoid confusion and allows preterm service to triage woman, who may only need additional CL scan and referral for appointment if cervix is short.
		Data validation added to the baby's date of birth on the POS.	To reduce errors by preventing input of implausible dates.
		PF-A to allow only moderate or high result for women with a BMI >40.	In response to user feedback. HCPs felt additional care should always be offered, even if risk=low. Also, for ease of alignment with any local high BMI guidance.
		Local admin user or System admin can export data directly.	To allow easy access to data for local and national audit and reporting purposes.
		HCP users can rerun a PTB assessment and a PF assessment after 20 weeks' gestation, e.g. if a data entry error has been identified, providing no later assessments have been run. Appropriate warnings will alert them to important information when doing so.	This is to ensure that any data entry errors identified can be corrected, and accurate assessment results can be confirmed.
		HCPs can save a draft of the ToB assessment allowing data entry without running the assessment. They can then return to the ToB assessment later add more data inputs and re-run later.	This allows the inputs to be entered as and when available, saving time when the actual assessment is run.
		The appearance of each different platform live, staging and training) has been changed to ensure that it is easy to distinguish between the platforms in use.	HCP users, Local admin user or System admin users can easily differentiate between the live/staging/training platforms.

		An additional warning appears to remind HCP users to check the accuracy of the medical history before running the PTB assessment.	Reduce errors that can lead to incorrect risk assessment results and care recommendations.
		New training platform optimised and ready for launch to all HCP users. Includes pre-populated records with dummy data to enable HCP users to simulate use of the live device.	For training/learning purposes (refresher training or new staff training) and for running dummy data to sense check a result where required for reassurance.
		HCP users can view the care pathway of a previous pregnancy for all pregnant users attending for care at their hospital. Pregnant users can view their own previous pregnancy details on their profile.	Tool record of previous pregnancy now available.
		HCPs are prompted to enter data into three additional fields on the medical history section to improve the PTB assessment.	To bring the PTB assessment in line with national guidance (SBLCBv3).
		Update to wording of autoimmune question in medical history section.	To make the data entry requirement clear and simple and avoid confusion.
		Local admin users receive an e-mail alert if anyone who reaches 38 weeks' gestation with either a moderate or high chance PFA result has not yet had their ToB assessment.	A mechanism to ensure that care recommendations offered are followed up.
		Pregnant users are notified through email about upcoming (gestation specific) care recommendations	Following consultation with the women's advisory group, gestation specific email alerts will be sent to pregnant users to alert them when care recommendations offered should be followed up.
		Extra fields added to data exports.	In response to user feedback.
		Functionality to will not allow creation of new account if at least two identifiers match.	To mitigate the erroneous creation of unnecessary duplicate profiles.
		The landing page updated to display distinct entry points for HCP users and pregnant users.	For clarification and user experience improvement and reduction of HCPs inadvertently registering for a pregnant user account.
		Branding, format and content of the landing page updated and the information behind each user page updated.	In response to user feedback and to improve user experience.
		Automated testing update.	Not visible to front end users (HCP Users) - improved automated testing.

1.8	Dec 2023	Data validation added to postcode data field.	To reduce errors.
		Banners added to the training site.	To ensure HCPs do not enter live data into the testing site by mistake.
		Data validation added to POS birth weight data field.	To prevent implausible birth weights from being entered.
		HCP interface: access to the Information Hub.	Previously, the Information Hub was only accessible to pregnant users. This increases awareness and reassures HCPs the information sources are reliable.
		PF-A now allows entry of individual test dates (scan, BP measurement, etc) in additional to date the assessment is run.	To reduce error.
		ToB-A and PPTL-A: addition of data points to record GDM or hypertension in pregnancy at time of assessment.	For more accurate data collection and allocation to correct care pathways.
		Facility to update pregnant user email address. User will be invited to re-verify account.	To allow pregnant user to regain access to account with different email address.
		Improved search function.	In response to user feedback.
		POS questions adjusted if pregnant user is transferred to another hospital.	To improve efficiency and reduce staff burden.
		Facility to allow NHS England administrator access to Tool data.	To improve reporting functionality for NHS England.
		Questions about previous pregnancies and medical conditions have been rephrased and reordered.	In response to user feedback; to prevent data entry errors and improve user experience.
		Facility to download a pdf of the care pathway overview and details of previous assessments.	HCPs can download results of PF-A and PTB-A with timestamp and demographic details. Particularly useful for hospitals using paper notes.
		HCP registration allows “NHS” email as well as nhs.net email address.	Not all HCPs have an NHS.net email address.
		Explanatory message if EDD from Tool and local scan reports do not match.	To reassure woman that a discrepancy of a couple of days will not negatively affect her care pathway.
		Amendment to POS questions.	To reduce burden and improve efficiency.
		Function to allow only scan reports after 36 weeks to be used in the ToB-A.	To reduce errors. ToB assessment is only accurate when entering data from scans conducted on or after 36+0 weeks' gestation.
		PPTL-A refined, irrelevant fields disabled, quantitative % risk shown as well as above/below 5% threshold.	In response to user feedback. To improve clarity and user experience.

		Answer format amended from dropdowns to yes/no buttons.	In response to user feedback. To improve user experience and reduce burden.
		Baby's NHS number on previous pregnancy POS hidden.	For data protection when birth parent and baby are not linked legally (surrogate, foster care, etc).
		POS: text defining pregnancy and postpartum hypertension added.	For clarity and to reduce errors.
		ToB-A: text added explaining reason for recommendation for women with high-risk PF-A result.	For clarity. Women with high PF-A result will be recommended IOL at 40 weeks as a minimum, regardless of ToB-A result.

**minor bug fixes not listed.*
