# Supplementary material for: Tommy’s Clinical Decision Support Tool: an intervention development and feasibility study to inform a future randomised controlled trial
Source: Pilot Feasibility Stud. 2026 Feb 25;12:45. doi: 10.1186/s40814-026-01788-9 (PMC13041263; doi:10.1186/s40814-026-01788-9)
Supplement: Supplementary file 4 — Additional file 4: “Adaptations to Implementation and training strategies during early adopter implementation” List of adaptations to implementation and training strategies with rationale for adaptation [file 40814_2026_1788_MOESM4_ESM.pdf]

Additional file 3: Adaptations to Implementation and training strategies during early adopter implementation

Year (Quarter)	Adaptations	Rationale
Adaptations to implementation strategy		
2021 (Q2)	Team meetings – days/times adapted to suit most local leads/champions.	To encourage more HCPs to attend meetings.
2021 (Q2)	Letter composed from RCOG and RCM and sent to Clinical Directors.	To confirm Royal Colleges' support, define project as service improvement and outline potential benefits.
2021 (Q3)	Appointment of WTE Tommy's Team Practical Implementation midwife to support early adopters in site set up and training.	To support sites and provide co-ordinated approach to staff training.
2021 (Q3)	Consideration to reallocate time from 2nd to 1st trimester scan (5 mins) to allow UADs. This would be in lieu of time saved when fewer growth scans will be needed as fewer women should be incorrectly identified as at high risk of placenta dysfunction.	Data on number of growth scans in Trust A & B appears to confirm this (albeit following an increase while the new system became embedded and confidence in the Tool grew).
2021 (Q3)	Admin, support workers, volunteers engaged to remind women and help to register on arrival if not yet done so.	To raise awareness and encourage more women to register ahead of their booking appointment.
2021(Q3)	Trust A using stickers to identify women registered on Tommy's App (based on sonographer asking at 1st trimester scan).	
2021 (Q3)	Tommy's Centre Clinical Director attended early adopter clinical meetings.	To promote project to staff and answer questions.
2021 (Q4)	Sharing of guideline addendums between sites (showing how Tool fits within current/most recent guideline and highlight where it differs)	To reduce burden for other early adopters and make implementation easier.
2021 (Q4)	Sharing strategies for change in practice, e.g. moving UAD to 1st trimester scan; aspirin PGD for midwives; PAPP-A for all women.	To reduce burden for other early adopters and make implementation easier.
2021 (Q4)	Large banners created and displayed in antenatal clinics.	To raise awareness and encourage more women to register ahead of their booking appointment.
2021 (Q4)	All maternity service users offered UAD at 1st trimester scan.	To avoid confusion and need for different explanations and consents from those booked for maternity care prior to Tool launch.
2022 (Q1)	Time from invitation email to verification of HCP accounts extended (as many had expired before HCP attempted to activate).	To prevent HCPs being "timed-out" before they verified their account.

2022 (Q1)	Local champions identified to cover different settings, i.e. community, antenatal clinic, labour ward, rather than just professional group.	To provide onsite and more timely support for staff when needed.
2022 (Q1)	Communications improved to ensure everyone understood that the Tool was now standard care, and not optional method of risk assessment.	Feedback indicated some believed women could "opt out".
2022 (Q2)	TNCfMI team starts referring to the Tool, in documentation and language, as "Tommy's Clinical Decision Support Tool" and "Tommy's Pathway", rather than "Tommy's App"	Reports from women and HCPs that users are searching for Tool on mobile app stores and are frustrated that "it's not an app".
2022 (Q2)	Information about forthcoming launch to be released earlier (decision taken that it was better to have some disappointed as booking too early, rather than so many women un-registered at booking)	To raise awareness and encourage more women to register ahead of their booking appointment.
2022 (Q2)	Lead midwife identified (Trust C) to take responsibility for implementation, training, risk, audit [where/when did this happen]	As midwives are the first, and most prolific users the Tool, it makes sense to have a midwife lead who is closer to this group of professionals and the potential concerns and issues they may encounter.
2022 (Q3)	Dedicated admin support employed by Trust C to audit bookings and women's registrations and follow up if not registered before booking appointment	To encourage more women to register ahead of their booking appointment, thus saving midwife time.
2022 (Q3)	Document detailing the role of the champion developed and distributed.	To clarify scope and role champions and to encourage new staff to take over when current champion moved on.
2022 (Q4)	Tool functionality was developed to allow champions to be "local admins", allowing them to add new staff as they are appointed, and deal with simple technical issues.	To allow faster responses to local issues
2022 (Q3)	Tool functionality introduced to allow HCP only registration.	To ensure those not able or willing to engage with Tool themselves are still able to have Tool risk assessments and recommended care pathways.
2024 (Q1)	Countdown to Launch document updated	To increase speed of readiness for launch
2024 (Q1)	Revised implementation toolkit (more concise, easier to navigate) including comprehensive FAQ document, based on questions received over entire early adopter implementation period.	Quicker and easier to access the information needed.

Adaptations to training strategy		
2021 (Q3)	Appointment of WTE Tommy's Team Practical Implementation midwife to support early adopters in site set up and training.	To support sites and provide co-ordinated approach to staff training.
2022 (Q3)	Cervical surgery guide developed by one and shared between sites.	To assist booking midwives undertaking PTB-A; to reduce number of women being assessed as high risk and referred to PTB service inappropriately
2021 (Q4)	USS training booklet developed by one and shared between sites.	To increase sonographer competence and confidence in undertaking uterine artery dopplers in first, rather than second, trimester.
2021 (Q3)	USS Tsar appointed to support USS department staff during the implementation.	To support early adopter site sonographer teams in training and increase confidence in new practice.
2022 (Q4)	Quick start guides/posters developed by one and shared between sites, improve HCP information resources	To increase staff confidence as they become familiar with new practice.
2022 (Q2)	Training plan developed – outlines training needs and timing by staff group.	To reassure sites that not all HCPs need to be trained prior to launch, and to stagger training so staff are not trained until just before they start using the Tool.
2022 (Q2)	Cascade training programme instigated by Practical Implementation Midwife.	To ensure training is carried out locally by individuals committed to the project.
2022 (Q2)	Local champions identified and given one-to-one training and then given access to staging platform for training purposes.	They could also check outcomes in cases of errors prior to training site availability and functionality to rerun assessments.
2023 (Q3)	Dedicated Training platform launched (easy access; pre-defined scenarios available).	To improve experience and accessibility of training.
2024 (Q1)	Training video updated.	To accommodate new features since launch.
2023 (Q4)	Training resources in implementation toolkit revised.	To accommodate new features since launch.
2024 (Q2)	Implementation toolkit updated (and will be made available on a website rather than behind Trust firewalls at start of RCT).	To accommodate new features since launch, to streamline, and to make it more accessible.
